# Neutrophil-to-lymphocyte ratio as a predictive or prognostic factor for gastric cancer treated with nivolumab: a multicenter retrospective study

**DOI:** 10.18632/oncotarget.26145

**Published:** 2018-10-02

**Authors:** Takatsugu Ogata, Hironaga Satake, Misato Ogata, Yukimasa Hatachi, Kentaro Inoue, Madoka Hamada, Hisateru Yasui

**Affiliations:** ^1^ Department of Medical Oncology, Kobe City Medical Center General Hospital, Chuo-ku, Kobe City, Hyogo, 650-0047, Japan; ^2^ Cancer Treatment Center, Kansai Medical University Hospital, Shinmachi, Hirakata City, Osaka, 573-1191, Japan; ^3^ Department of Surgery, Kansai Medical University Hospital, Shinmachi, Hirakata City, Osaka, 573-1191, Japan

**Keywords:** advanced gastric cancer, nivolumab, neutrophil-to-lymphocyte ratio, predictive factors, prognostic factors

## Abstract

**Introduction:**

The neutrophil-to-lymphocyte ratio (NLR) is effective as a predictive factor for lung cancer treated with nivolumab. The objective of this study was to determine the effectiveness of NLR for patients with advanced gastric cancer (AGC) treated with nivolumab.

**Methods:**

This was a multicenter, retrospective study of patients with AGC treated with nivolumab from June 2017 to December 2017. The NLRs were calculated before the first cycle (NLR_pre_) and two weeks after the first administration (NLR_post_).

**Results:**

Twenty-six patients were enrolled (males 19, females 7) with a median age of 64 years. The overall response rate was 15%. The median PFS was 80 days (range, 11 – 265) and the median OS was 290 days (range, 21 – 332). Stratified with high NLR (≥5) and low NLR (<5), the median PFS was shorter in the high NLR_pre_ arm (87 vs. 45 days; p=0.066) and significantly shorter in the high NLR_post_ arm (94 vs. 28 days; p=0.014). The median OS was significantly shorter in the high NLR_pre_ arm (290 vs. 175 days; p=0.008) and in the high NLR_post_ arm (290 vs. 69 days; p<0.001).

**Conclusion:**

NLR may be an effective prognostic factor in patients with AGC treated with nivolumab.

## INTRODUCTION

Immune checkpoint inhibitors are now widely used for many kinds of tumors. In 2017, the ATTRACTION-2 study [1] showed that nivolumab monotherapy was more effective for the pretreatment patients with advanced gastric cancer (AGC) than a placebo, and that it can be used in clinical practice for treatment [2]. However, in approximately 50% of patients, nivolumab was effective, and in only 10% of patients, nivolumab remained effective for more than a year according to the ATTRACTION-2 study [1]. In light of the more favorable responses observed in patients treated with nivolumab, many studies have focused on identifying predictive factors for favorable nivolumab responses. For example, programmed death ligand-1 (PD-L1) or programmed death ligand-2 (PD-L2), mutation burden, and mismatch repair deficiency (dMMR), have been investigated in many studies in terms of their association with nivolumab [3–6]. However, measuring these factors is not easy and can be time consuming. The pretreatment neutrophil-to-lymphocyte ratio (NLR), a marker of systemic inflammation, was recently shown to be associated with outcomes in a variety of cancers [3, 4]. Some studies have reported that NLR is an effective prognostic and predictive factor. In patients with gastric cancer, Tanaka et al. reported that NLR at the time of diagnosis reflects the progression of metastasis [5], and Min et al. reported that the postoperative NLR was associated with patient survival [6]. In patients with lung cancer treated with nivolumab, NLR was also associated with progression free survival (PFS) or overall survival (OS) [7, 8]. Kiriu et al. reported that NLR at 2 weeks after the first administration of nivolumab was associated with time to treatment failure [9]. However, the effectiveness of NLR as a prognosticator among patients with AGC treated with nivolumab is unknown. The objective of this study was to determine the effectiveness of NLR for AGC treated with nivolumab monotherapy.

## RESULTS

### Patient characteristics

Twenty-six patients were treated with nivolumab for AGC. Patient characteristics are summarized in Table 1. The median age at the time of treatment was 64 years (range, 44 – 86; interquartile range [IQR], 59 – 71), and 73% of the patients were male. The median pretreatment NLR (NLR_pre_) was 2.22 (range, 0.94 – 17.4; IQR, 1.52 – 4.57), with the NLR_pre_ < 5 in 20 patients (77%) and NLR_pre_ ≥ 5 in 6 patients (23%). The median posttreatment NLR (NLR_post_) was 2.84 (range, 1.03 – 12.3; IQR, 1.98 – 3.81), with NLR_post_ < 5 in 21 patients (81%) and NLR_post_ ≥ 5 in 5 patients (19%).

**Table 1:** Patient characteristics

	N = 26	(%)
Gender, male/female	19/7	(73/27)
Age, median (range)	64	(44 - 86)
ECOG PS, 0/1/2	2/22/2	(8/85/8)
Location, Upper/Middle/Lower	9/9/8	(35/35/31)
Lauren classification, Intestinal/Diffuse	14/13	(52/48)
HER2 status, +/-	3/23	(12/89)
Number of previous treatments, 1/2/≥3	3/11/12	(12/42/46)
Previous radiotherapy, +/-	3/23	(12/89)
Liver metastasis		
Yes/No	7/19	(27/73)
Ascites		
Yes/No	14/12	(54/46)
Pretreatment NLR, median (range)	2.22	(0.94–17.4)
Pretreatment NLR, <5/≥5	20/6	(77/23)
Posttreatment NLR, median (range)	2.84	(1.03–12.3)
Posttreatment NLR, <5/≥5	21/5	(81/19)

Abbreviations: ECOG PS, the Eastern Cooperative Oncology Group Performance Status; NLR, neutrophil-to-lymphocyte ratio.

### Response to the treatment

The overall response rate was 15% (4 of 26 patients), and the overall disease control rate was 42% (11 of 26 patients). When stratified according to high NLR (≥5) and low NLR (<5) values, the response rates were not significantly different (Table 2).

**Table 2:** NLR levels of response

		CR	PR	SD	PD
NLR_pre_	high	0	0	1	5
	low	1	3	6	10
NLR_post_	high	0	0	0	5
	low	1	3	7	10

Abbreviations: CR, complete response; PR, partial response; SD, stable disease; PD, progressive disease.

### Progression free survival and overall survival

**Figure 1:**
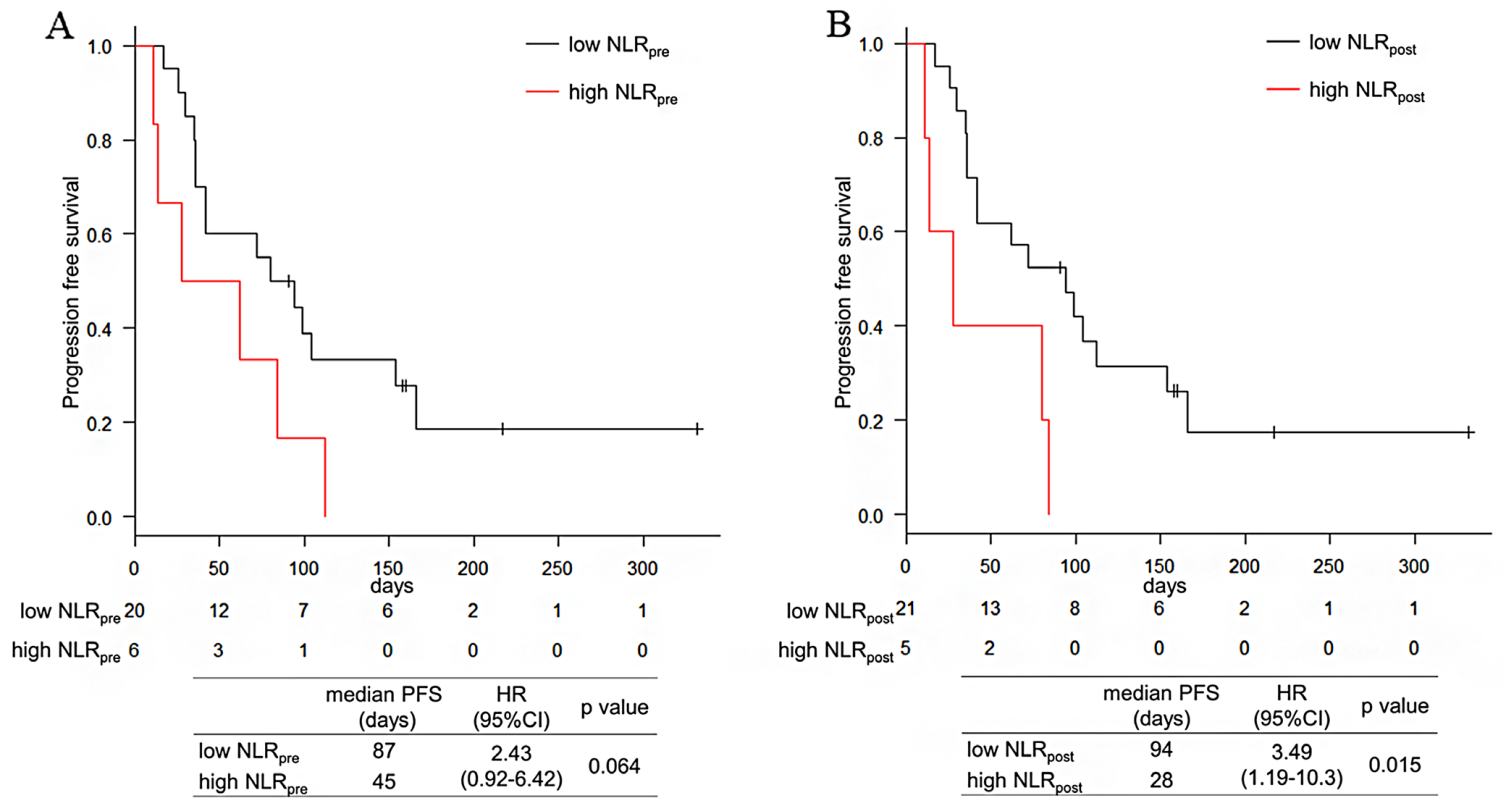
Progression free survival of NLR_pre_ and NLR_post_ **(A)** Kaplan–Meier survival curve according to the NLR_pre_. **(B)** Kaplan–Meier survival curve according to the NLR_post_.

**Figure 2:**
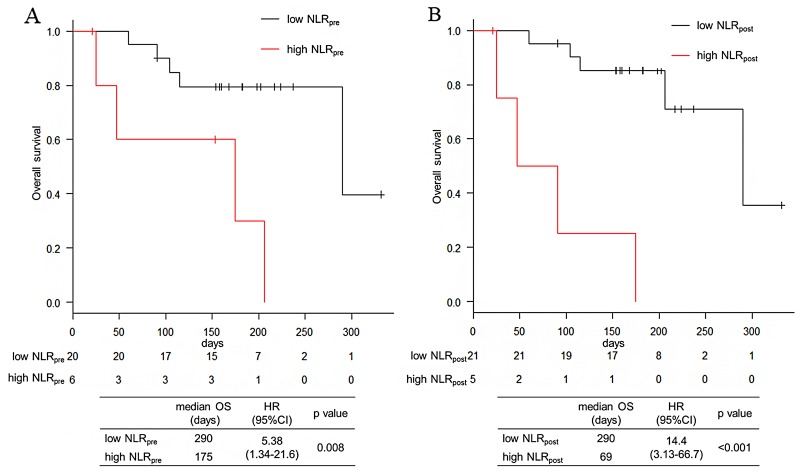
Overall survival of NLR_pre_ and NLR_post_ **(A)** Kaplan–Meier survival curve according to the NLR_pre_. **(B)** Kaplan–Meier survival curve according to the NLR_post_.

With a median follow up of 171 days, the median PFS was 76 days and the median OS was 290 days in the total population. The median PFS tended to be shorter in the high NLR_pre_ arm (87 days vs. 45 days; hazard ratio [HR] 2.43; 95% confidence interval [CI]: 0.92 – 6.42; p=0.064) and significantly shorter in the high NLR_post_ arm (94 days vs. 28 days; HR 3.49, 95% CI: 1.19 – 10.3; p=0.015) (Figure 1). The median OS was also significantly shorter in the high NLR_pre_ arm (290 days vs. 175 days; HR 5.38, 95% CI: 1.34 – 21.6; p=0.008) and in the high NLR_post_ arm (290 days vs. 69 days; HR 14.4, 95% CI: 3.13 – 66.7; p<0.001) (Figure 2). The other factors were not associated with PFS and OS (Table 3).

**Table 3:** Univariable analyses for progression free survival (PFS) and overall survival (OS)

PFS:				
		HR	95%CI	p value
Gender	Male/Female	1.66	0.60–4.63	0.322
Age	≥65/<65	0.638	0.26–1.55	0.314
ECOG PS	0/1/2	2.20	0.80–6.04	0.251
Tumor location	U/L	1.84	0.58–5.85	0.302
	M/L	2.79	0.88–8.83	0.303
Lauren classification	Intestinal/Diffuse	1.25	0.53–2.95	0.603
HER2	+/-	2.46	0.71–8.59	0.142
Liver metastasis	+/-	0.84	0.32–2.22	0.724
Ascites	+/-	1.04	0.44–2.48	0.926
Pretreatment NLR	≥5/<5	2.43	0.92–6.42	0.064
Posttreatment NLR	≥5/<5	3.49	1.19–10.3	0.015

OS:				
		HR	95%CI	p value
Gender	Male/Female	2.23	0.45–11.0	0.310
Age	≥65/<65	1.47	0.39–5.57	0.565
ECOG PS	0/1/2	4.05	0.87–18.8	0.172
Tumor location	U/L	2.33	0.24–23.1	0.469
	M/L	3.51	0.40–30.5	0.256
Lauren classification	Intestinal/Diffuse	1.23	0.29–5.16	0.779
HER2	+/-	–	–	–
Liver metastasis	+/-	–	–	–
Ascites	+/-	1.54	0.38–6.20	0.538
Pretreatment NLR	≥5/<5	5.38	1.34–21.6	0.008
Posttreatment NLR	≥5/<5	14.4	3.13–66.7	<0.001

Abbreviations: ECOG PS, the Eastern Cooperative Oncology Group Performance Status; U, Upper; M, Middle; L, Lower.

### Immune-related adverse events

Regarding immune-related adverse events, hypothyroidism occurred in 2 patients, elevated liver enzyme levels in 2 patients (grades 1 and 3), and vitiligo in 1 patient. One patient with grade 3 elevated liver enzyme level discontinued treatment. The four patients with hypothyroidism, elevated liver enzyme levels, and vitiligo continued receiving treatment after the occurrence of immune-related adverse events.

## DISCUSSION

We observed that NLR, especially NLR_post_, was associated with PFS and OS. Our results indicated that the NLR might be useful as a prognostic factor of treatment with nivolumab monotherapy for AGC. This is the first report to show an association between NLR and clinical outcomes including PFS and OS for advanced gastric cancer patients treated with nivolumab.

We evaluated tumor responses and progressive disease according to RECIST and we considered the change in treatment. However, the activities of immune checkpoint inhibitors (ICIs), are different from cytotoxic agents, and pseudoprogression and hyperprogressive disease have been reported in patients treated with ICIs. In gastric cancer, hyperprogressive disease has been previously reported [10]. When the pseudoprogression occurred, we considered a change in treatment according to RECIST because we could not decide whether the progression was pseudoprogression or hyperprogressive disease. Recently, iRECIST was advocated [11], and “beyond progression” has been increasingly considered in tumor progression in treatment with ICIs. According to iRECIST, pseudoprogression may be noticed, but in the case of hyperprogressive disease the treatment is often continued inadequately. However, from the results of this study, it might be necessary to consider changing drugs as soon as progression is noted, when the NLR is high.

For the PFS and OS, the high NLR_pre_ tended to be short. Since nivolumab monotherapy for AGC is considered the salvage line, nivolumab monotherapy will be selected as the third line in many cases even if the NLR_pre_ is high. The hazard ratios of PFS and OS in NLR_post_ were higher, which was thought to have greater effects on PFS and OS. Therefore, it is better to consider changing the regimen before progression, when NLR_post_ exceeds the threshold of 5 and they were treated with nivolumab; therefore, the study of Jung et al. [12] and the study of NSCLC [7–9] that included treatment with nivolumab were referred to for the NLR cut-off value (Table 4). Nivolumab was different from the other cytotoxic agents, so nivolumab monotherapy for NSCLC was used as the reference. It is necessary to discuss whether this cut-off value is valid or not, but it was suggested that NLR could predict survival.

**Table 4:** Studies of the baseline NLR for AGC treated with chemotherapy

a) Studies on the efficacy of NLR for evaluating AGC					
Treatment	Patients	Cut-off	Line	Outcome	Reference
RAM±PTX	265	5	2^nd^	PFS, OS	[12]
Chemotherapy	104	3	1^st^	OS	[21]
Chemotherapy	268	3	1^st^	OS	[22]
S-1+CDDP	110	3	1^st^	OS	[23]
Chemotherapy	868	2.7	2^nd^	OS	[24]
Chemotherapy	143	3.34	1^st^	PFS, OS	[25]

b) Studies of the efficacy of the NLR for NSCLC treated with nivolumab					
	Patients	Cut-off	Line	Outcome	Reference
	175	5	2^nd^–	OS	[7]
	19	5	2^nd^–	PFS, OS	[9]
	52	5	—	PFS, OS	[8]

Abbreviations: PFS, progression free survival; OS, overall survival; RAM, ramucirumab; PTX, paclitaxel; CDDP, cisplatin.

The mechanism underlying the association of NLR_post_ with PFS or OS is not clearly understood. We hypothesized the influence of interleukin (IL)-17. It has been reported that IL-17 release the substance that mediates the migration of neutrophils [13]. In addition, IL-17A has been reported to be involved in the mechanisms of resistance to immune checkpoint inhibitors [14]. In the case of high NLR_post_, the NLR was elevated because IL-17 levels had not decreased after the first dose of nivolumab.

### The biomarkers of immune checkpoint inhibitors

In many studies regarding ICIs, biomarkers have been investigated. The expression of PD-L1 or PD-L2 [15], mutation burden [16], dMMR [17] are said to be associated with the efficacy of ICIs. Furthermore, the expressions of PD-L1 and PD-L2 are associated with the response to ICIs [15]. In patients with lung cancer, pembrolizumab was more effective for higher expression of PD-L1 [18]. However, in patients with gastric cancer, the rate of positivity for PD-L1 (>1%) was only 17.3% [15]. When somatic mutation burdens of melanoma and non-small cell lung cancer were high [19], then the efficacy of ICIs was very high [16]. On the other hand, mutation burdens of gastric cancer were not very high [19]. The dMMR is reported to be a predictive factor of ICIs, and has been identified in 27% of patients with gastric cancer [20]. Identification of these biomarkers involved the use of archival specimen, and so did not reflect the current status, especially the third line. However, the NLR is easily identified at the time of drawing blood and dynamic changes can also be identified.

### The limitation of this study

The limitations of this study include its retrospective design, the small cohort size, and the relatively short follow up period. However, the overall response rate was 12% and the disease control rate was 42%. These results were similar to those of the ATTRACTION-2 study [1], which is one reason why our study was valid regardless of the retrospective design that included only two centers. However, because of the relatively small cohort size, a multivariate analysis was not possible. It is, therefore, necessary to perform a similar larger analysis using a prospective design to confirm the results. The follow up period of this study was relatively short, and therefore we will continue to follow the patients and report the updated data.

In conclusion, NLR, especially posttreatment NLR, may be effective as a prognostic factor in patients with gastric cancer treated with nivolumab monotherapy. Further studies are warranted to investigate how NLR can be used to detect disease progression as early as possible.

## MATERIALS AND METHODS

### Patients and treatments

We performed a retrospective study based on the medical records of patients at two centers, Kobe City Medical Center General Hospital and Kansai Medical University Hospital. The patients with gastric cancer were histologically diagnosed with adenocarcinoma, and they all had experienced either recurrence after surgery or developed de novo Stage IV cancer. Patients included in the study were treated with nivolumab monotherapy. The data collected included the following: gender, age, Eastern Cooperative Oncology Group Performance Status (ECOG PS), tumor location, HER2 status, prior treatment histories at the time of the first administration of nivolumab, the history of radiotherapy, the location of metastasis, NLR_pre_, and NLR_post_. Nivolumab was administered intravenously at dose of 3 mg/kg as one cycle every two weeks.

This retrospective analysis was approved by the Institutional Review Board of Kobe City Medical Center General Hospital and Kansai Medical University Hospital.

### Neutrophil-to-lymphocyte ratio

We recorded absolute neutrophil count (ANC) and absolute lymphocyte count (ALC) on the day of the first administration and also two weeks after this. The NLR was defined as ANC divided by ALC. The NLR_pre_ was defined as the NLR at the time of the first administration, and NLR_post_ was defined as the NLR at two weeks after the first administration of nivolumab.

A previous study found that a baseline NLR < 5, which is commonly used as an NLR threshold, was associated with an improved survival in lung cancer patients treated with nivolumab [7], and in gastric cancer patients treated with paclitaxel and ramucirumab therapy [12]. We, therefore, used 5 as the NLR cut-off value in this study.

### Statistical analysis

OS was defined as the time from the date of first administration of nivolumab to the date of death due to any cause. Patients who were still alive were censored at the last follow-up. PFS was defined as the time from the date of first administration of nivolumab until disease progression. The OS and PFS curves were estimated using the Kaplan–Meier method and compared using the log-rank test. P < 0.05 was considered statistically significant in all analyses. The analysis was performed using R (The R Development Core Team, Vienna, Austria).

## Abbreviations

AGC: advanced gastric cancer
ALC: absolute lymphocyte count
ANC: absolute neutrophil count
CI: confidence interval
dMMR: mismatch repair deficiency
ECOG PS: Eastern Cooperative Oncology Group Performance Status
HR: hazard ratio
IQR: interquartile range
NLR: neutrophil-to-lymphocyte ratio
NLR_post_: posttreatment NLR
NLR_pre_: pretreatment NLR
OS: overall survival
PD–L1: programmed death ligand–1
PD–L2: programmed death ligand–2
PFS: progression free survival
